# Supplementary material for: Photoinduced DNA Cleavage and Photocytotoxic of Phenanthroline-Based Ligand Ruthenium Compounds
Source: Molecules. 2021 Jun 7;26(11):3471. doi: 10.3390/molecules26113471 (PMC8201372; doi:10.3390/molecules26113471)
Supplement: Supplementary file 1 [file molecules-26-03471-s001.zip › molecules-1204857-supplementary.pdf]

Supplementary material

molecules

Photoinduced DNA Cleavage and Photocytotoxic of Phenanthroline-Based Ligand Ruthenium Compounds

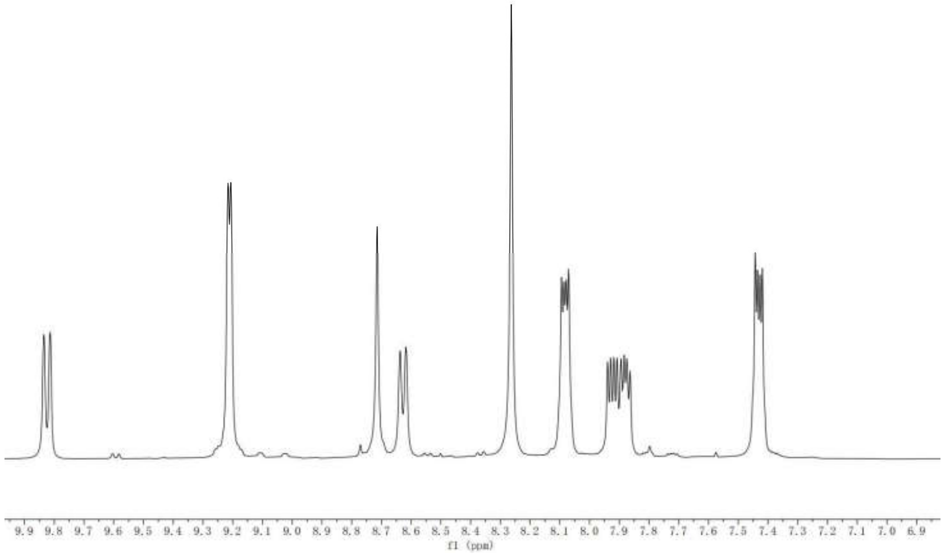

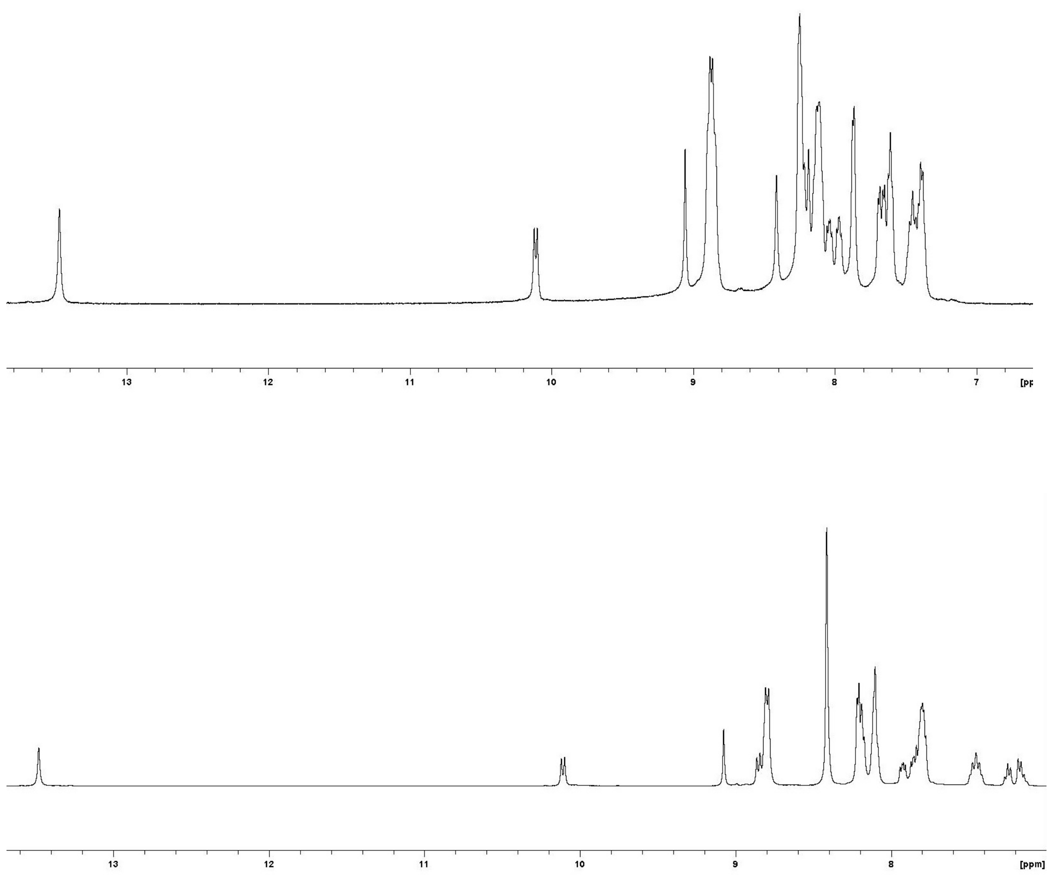

Figure S1.^{1}H NMR in aromatic region of the ligand pni(top), complex **1** (middle) and **2** (bottom) in $(CD_3)_2SO$ (400 MHz)

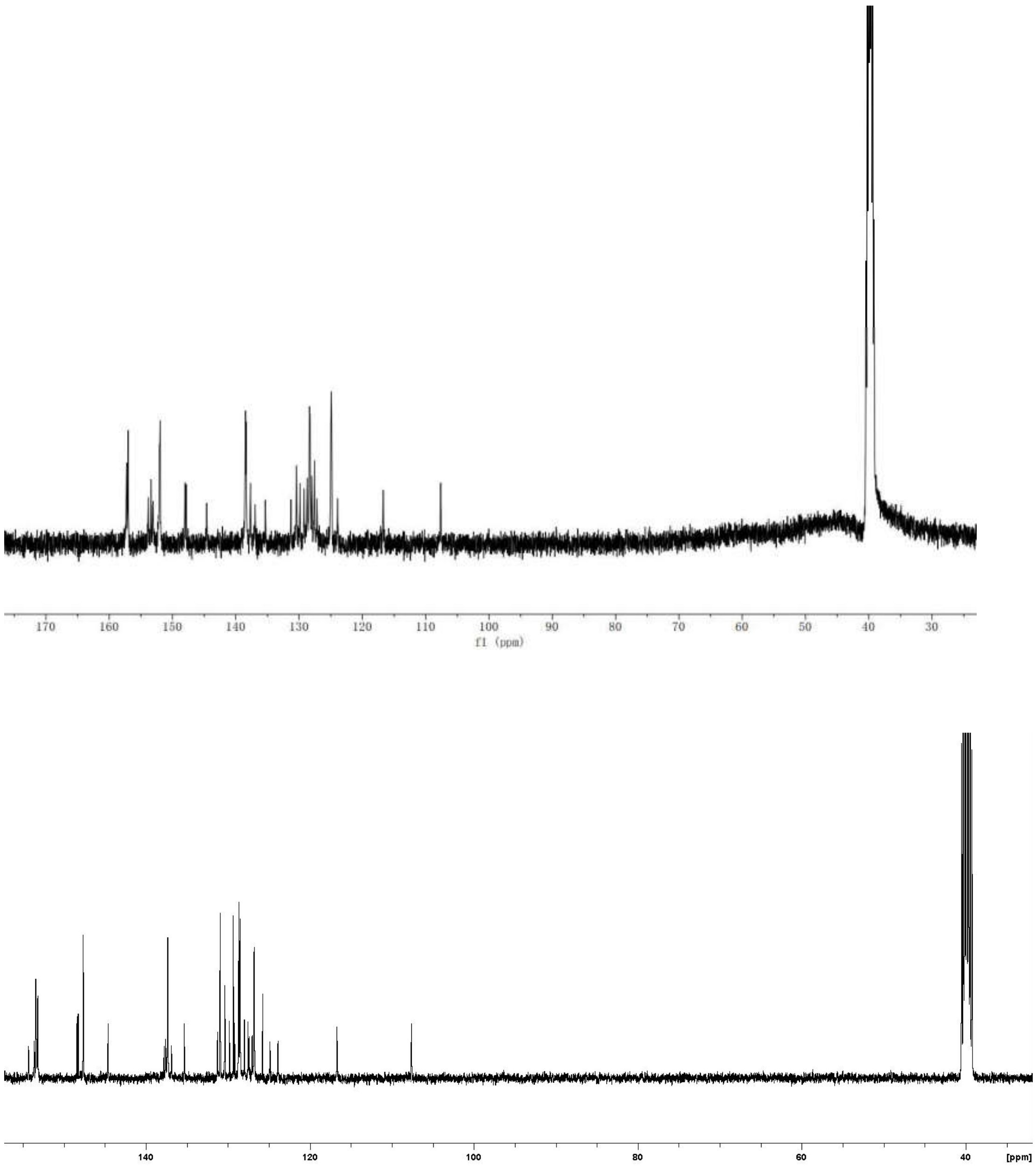

Figure S2. ^{13}C NMR in aromatic region of complex **1** (top) and **2** (bottom) in $(CD_3)_2SO$ (100 MHz)

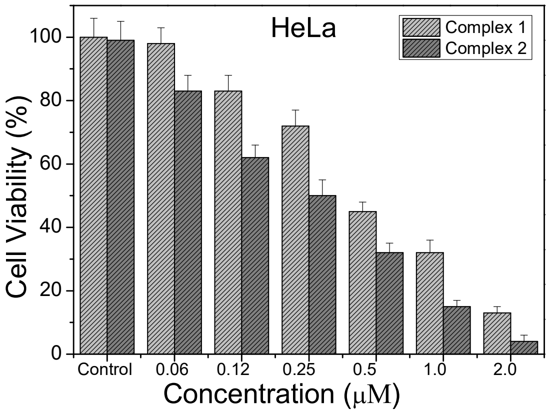

Figure S3. Cell viabilities of HeLa cells pretreated with various concentrations complexes **1** and **2** for 12h and irradiated at 450 nm,for 10 min (6 mW/cm^2).

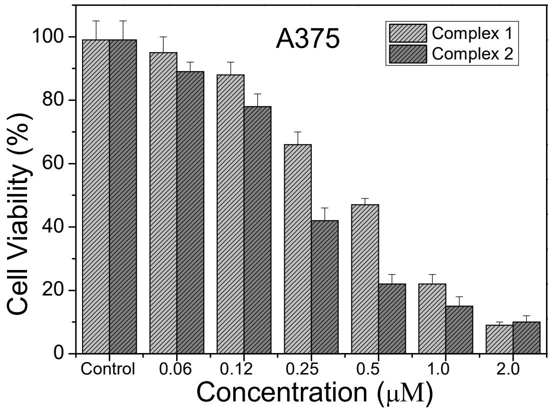

Figure S4. Cell viabilities of A375 cells pretreated with various concentrations complexes **1** and **2** for 12h and irradiated at 450 nm,for 10 min (6 mW/cm^2).

Singlet Oxygen Quantum Yield Measurement

The $^1O_2$ generation quantum yields of Ru(II) complexes were measured by reaction between 1,3-diphenylisobenzofuran (DPBF) with singlet oxygen. The 2 ml air-saturated methanol solutions containing DPBF (20 μM) and complexes (20 μM) were illuminated with light of 450 nm (obtained from Hitachi F-2500 spectrofluorophotometer, 5 nm of excitation slit width). The consumptions of DPBF

were monitored its fluorescence intensity decrease at the emission maximum (λ_{ex} = 405 nm, λ_{em} = 479 nm) at different irradiation time.

The $^1O_2$ generation quantum yield (Φ_Δ) was calculated according to eq. S1 and S2, where I_{in} is the incident monochromatic light intensity, Φ_{ab} is the light absorbing efficiency of the photosensitizer, Φ_r is the reaction quantum yield of $^1O_2$ with DPBF, t is the irradiation time, I_0 and I_t is the fluorescence intensity of DPBF before and after irradiation, k is the slope, and superscript s stands for standard.

$$\frac{-\Delta[DPBF]}{t} = \frac{I_0 - I_t}{I_0} = I_{\mathrm{in}} \Phi_{\mathrm{ab}} \Phi_{\Delta} \Phi_{\mathrm{r}} \qquad \text{(S1)}$$

$$\frac{k}{k^s} = \frac{\Phi_{\mathrm{ab}}}{\Phi_{\mathrm{ab}}^{\mathrm{s}}} = \frac{\Phi_{\Delta}}{\Phi_{\Delta}^{\mathrm{s}}} \qquad \text{(S1)}$$
